# Communicable Diseases

**Published:** 1882-10

**Authors:** George E. Blackham


### COMMUNICABLE DISEASES.

GEO. E. BLACKHAM, M. D.

Diseases may be roughly divided into two classes—communicable and non-communicable. Communicable diseases are, of course, those which may be communicated from person to person, and in which, therefore, each person attacked becomes actually, or potentially, the center of an ever widening circle of disease. While there may be some diseases about which doubt may exist as to which class they belong in, there are a number about which no doubt can exist in the minds of people who have given the subject attention. Communicable diseases again may be divided into two sub-classes, viz.: *contagious* and *infectious*. *Contagious* diseases being those which are communicable only by direct *contact*, and *infectious* diseases being those in which the infection can operate through a greater or less distance. Of the former class the "Itch," and of the latter "Diphtheria" may be taken as characteristic examples, though it is more than probable that no distinct line of demarkation can be drawn between the two sub-classes except roughly and for mere practical convenience of description.

It will probably be found, if indeed it is not already demonstrated, that all communicable diseases are dependent upon the presence and growth in or upon some of the tissues of the patient of some parasitic organism, either animal or vegetable, which lives at the expense of the tissue in or upon which it is located, and also acts as a local irritant thus, in two distinct ways, interfering with the proper discharge of the functions, that is to say with health.

That this is the case with the disease known as "Scabies," or "The Itch" is now well known. It is produced by the "Itch Insect," so-called—a minute eight-legged *mite* which, burrowing under the skin of the patient, produces by its presence and tunnelling operations the peculiar eruption and intolerable itching so characteristic of the disease. The communication in this disease is *contagious* because the Itch mite, though small, is too heavy to float in the atmosphere, and consequently can only be transferred to a new patient when the latter is brought into actual physical contact with a person already suffering from the disease. Concerning the Itch-mite and its position as the essential factor, the *direct cause*, of the disease there is now no question. The parasite is large enough to be isolated and transplanted, and we can readily comprehend the way in which the disease effects are produced by the presence of tiny creatures crawling over the surface of the skin, and digging tunnels into it in which to deposit their eggs. No one, no educated physician at least, would now be willing to claim that the "Itch-mite" was a *consequence* and not the cause of the disease, or that by reason of some fancied "unity of disease" the the itch germs, when transferred to the skin of a new victim, might produce Itch, or some other disease, according to "the state of the system" of the patient. It is recognized that, like all other animals, they can bring forth only after their own kind—and that the progeny of the Itch-mite must be Itch-mites and nothing else—and that they can not arise *de novo*, by spontaneous generation, and hence, that every case of Itch demonstrates the previous existence of some other case of Itch, and that the parasite which causes the disease was transferred from the old to the new case.

When we come to consider cases of *infectious* disease, however, the subject becomes far less simple and easily understood, especially by persons not familiar with the use of the microscope and the progress of microscopical research. We have now to do with vegetable not animal parasites—organisms so minute that the Itch-mite is to one of them as Jumbo to a mosquito—and which in some cases tax the powers of our best microscopes and the skill of our best microscopists to define them at all, and which it is, therefore, very difficult to classify upon any basis, except that of the *effects* produced by them on the human system. To the believers in the "Germ Theory" of infectious diseases, however, the chain of reasoning seems clear and satisfactory, though many of the details of the life histories of these disease-producing plants remain to be investigated.

The disease cycle seems to be as follows: The disease plant having attained its growth gives off numerous spores, which are almost infinitely minute and so light that they float about in the air, like thistle seeds, till they find some resting place. If this be unfavorable for their development they may remain quiescent, but living, for an indefinite period of time. If, on the other hand, they find a favorable location, they immediately begin to develop into perfect plants and, in due time, reach their maturity and give off their spores to fill the atmosphere with disease germs and spread infection on every passing breeze. Those who accept the "germ theory" of disease, believe that scarlet fever, small-pox, diphtheria, measles, typhoid fever, etc., have each their own specific disease-producing parasitic plant, the growth of which is the true cause of those disturbances of function in the human system which constitute the particular disease belonging to that special parasitic plant.

Let us, in the light of this theory, trace the history of a case of diphtheria.

The patient, a child let us say, in traveling in the cars or elsewhere, is "exposed to the disease," that is to say, breathes air contaminated with the spores of the diphtheria plant. Some of them lodge in the mucous membrane of the throat, and after a period of incubation, the duration of which is not exactly known, and which probably varies somewhat with varying conditions, the patient begins "to feel badly." There is, perhaps, more or less headache, sore throat, furred tongue, loss of appetite, constipation, etc. Soon a "false membrane" appears in the throat, the febrile symptoms increase in severity, there is great depression, nervous symptoms supervene. If the "false membrane" extends into the windpipe the patient may choke to death, if it does not he may fall a victim to failure of the heart from oppression of the nervous centres, or to general debility, or there may be a gradual recovery.

Now, what has the diphtheria plant been doing all this while, and what relation do the various stages in its life history bear to the progress of the disease?

First, then, during the period of incubation, that is the period between exposure and the appearance of the first symptoms of sufficient importance to attract attention, the spores which have found lodgment on the mucous membrane of the air passages, have been growing, they have been developing into plants, sending down their minute threads of root and branch into the epethelial cells of the mucous membranes and sucking out and appropriating to their own uses the protoplasmic substances upon which the activity and usefulness of these cells depend; they have been multiplying with fearful rapidity, sending out their spores by myriads to invade new territory, loading the blood with them to be carried to distant parts of the body; to the brain to grow in and live upon the brain cells and interfere with their functions; to the muscles to produce muscular pain and weakness; to the bowels to produce interference with their functions and consequent constipation, etc. In short we have all the symptoms of a specific form of blood poisoning as regards the system at large. Meanwhile the original point of infection is still the "base of operations" of the invading army of parasitic plants; here they are most numerous and their local destructive effects most marked. The superficial cells of the mucous membrane of the throat having the life sucked out of them, so to speak, die in large numbers, and these dead and dying cells, becoming matted with the mycelium threads of the parasite, form the patches of the so-called false membrane.

The irritation resulting from the presence of a foreign body causes congestion and imflammation, and we find the mucous membrane of the throat darkened, congested, thickened and inflamed and covered with patches of "false membrane." In these dead and dying masses a new form of destructive change may take place, viz.: Putrescence, and we get the condition commonly known as putrid sore throat, in which the system is then subjected to the additional danger of secondary blood-poisoning from the absorption of putrid matter. If the patient's strength does not hold out he will succumb to some or all of these abnormal conditions, while on the other hand if suffocation can be prevented and the vital forces be sustained till the parasitic plant has exhausted the soil in which it grows the patient will gradually recover. This is the course in typical cases, and similar histories in which the parallelism of the life-histories of the disease, and the disease *plant* would appear could be given for other communicable diseases such as Small-Pox, Typhoid Fever, etc.; but this will suffice to illustrate the reasonableness of the theory.

But what is the use of all this theorizing, all this fine study of the life cycle of invisible vegetation, what practical lessons can we draw from it as to the prevention and cure of this class of diseases?

1st. Then we may learn the great value of careful sanitary measures, as it is probable that these noxious disease-plants may and do grow and send forth spores wherever they can find organic matter in a decaying condition. The neglected cesspool or drain which was merely, or chiefly, an offense to the nostrils becomes a disease center within a few hours after a few disease germs have found a lodgment in it.

2d. We may learn something of the general *principles* which should guide us in the treatment of this class of diseases. We should not pin our faith exclusively to either local or general measures, but use both, each in its proper place.

3d. We may learn that in these diseases the most rigid isolation should be observed. The house in which a patient lies ill of diphtheria, scarlet fever, or typhoid fever, should be as rigidly quarantined as if the case were one of small-pox or yellow fever. The patient should be visited only by those actually required to nurse and care for him, and they should have as little communication with the outside world as possible. All secretions, excretions or discharges of any kind from the patient should be disinfected before being taken from the sick room. In case of death, *the funeral should be strictly private.*

It is little, if any thing, less than criminal to gather a host of friends around the body of one who has fallen a victim to a disease of this class, keep them sitting for an hour or more in an atmosphere which for days and weeks, perhaps, has been filled with myriads upon myriads of disease-producing spores, where you can not touch the walls, or the carpet, or a piece of furniture without the probability of setting untold millions of these tiny spores flying and filling your own clothing and that of every one near you with them, to lie dormant for a time, and then, perhaps, to be transferred to some child or friend and cause a death.

No sentimental considerations should be permitted to interfere with our duty to the living. No dogmatic assertions of pompous funeral directors (in plain English undertakers), that *their* method of embalming, etc., removes all danger of infection, should tempt us to relax for an instant the vigilance with which we should guard against the spread of disease. The undertakers' art has doubtless made progress of late; by their skill the dangers of infection may be, and doubtless are, much decreased; but too much yet remains to make the assembling of friends around the body, in the house, or the transfer of the body to church, justifiable. The body of one dead from a communicable disease should be finally disposed of as quickly and as privately as possible. Then, if considered desirable to make a display of sorrow and sympathy, funeral services could be held in some church or other building, but *not in the house where the person died.*

After all is over the house should be thoroughly aired and disinfected throughout, while the sick-room should be thoroughly renovated; the old paper being removed *and burned;* the bed-clothes boiled, etc., and none but members of the household admitted to the house till after its purification is complete.

